# What Is the Mediating Role of Communication Skills and Sexual Satisfaction between Job and Life Satisfaction of Healthcare Employees?

**DOI:** 10.3390/bs13050368

**Published:** 2023-04-28

**Authors:** Sema Üstgörül, Catalin Popescu

**Affiliations:** 1 Faculty of Health Science, Manisa Celal Bayar University, Manisa 45140, Turkey; 2 Department of Business Administration, Petroleum-Gas University of Ploiesti, 100680 Ploiesti, Romania

**Keywords:** job satisfaction, life satisfaction, sexual satisfaction, communication skills, healthcare organization

## Abstract

There are three areas of harmony in human life that are related to each other: “work environment”, “love-to be loved-sexual area” and “social environment”. Incompatibility and dissatisfaction in one area can also affect other areas. Therefore, the aim of this study is to examine the relationship between job satisfaction, life satisfaction, communication, and sexual satisfaction of healthcare employees. The data collected by questionnaires from 394 employees working in university hospitals in Turkey were analysed using SPSS and AMOS programs. The findings show that there is a positive relationship between the job and life satisfaction of employees of healthcare organizations. Additionally, the findings revealed that communication skills and sexual satisfaction have a mediating role between job satisfaction and life satisfaction of employees in healthcare organizations. Life satisfaction, sexual satisfaction, and relationships are some of the factors that should be considered by healthcare organizations. It would be beneficial to employees and the public if health policy makers implemented programmes to enhance job satisfaction.

## 1. Introduction

When employees are more satisfied in their life and job, this in turn leads to a higher degree of both psychological and mental relaxation [1]. Therefore, satisfaction with business life is closely related to the ability of an employee to perform the functions listed [1]. Job satisfaction is more than just the satisfaction of employees with their jobs and whether their needs are met [2]. The Hierarchy of Needs theory states that the lowest need is to meet the needs of the body, such as food, drink, and sex, followed by the needs for security, belonging, acceptance, love, and self-actualization. According to Maslow’s Hierarchy of Needs, sex is one of the basic needs for a quality life [3]. Maslow’s hierarchy categorizes sex as a physiological requirement alongside eating and breathing; it lists sex only from an individualistic standpoint. For example, sex is grouped with other physiological requirements that must be met before considering “higher” levels of motivation [4]. Negative consequences for employees and organizations are caused by low job satisfaction, as there may be tensions caused by job dissatisfaction and related psychosomatic disorders. Working in harmony, having the need for prestige, and progress in the organization for self-actualization are examples [5].

People who are not satisfied with their jobs try to fulfil their duties by working with low efficiency and performance [6]. The main purpose of organizations is to benefit from the performance of employees. Obtaining a high level of efficiency from employees is important not only for the development of organizations but also for the development of society [7].

Competitive environments that require quality and cost efficiency are more successful for organizations that can create a work environment that attracts, motivates, and retains employees. Job satisfaction and performance have always been important issues for human resource management practitioners [8].

Organizations require satisfied and psychologically balanced employees to increase performance and productivity [9]. Healthcare employees in low-income countries are less satisfied with their jobs due to limited resources and poor job conditions [10]. Irregular working hours, sleep disorders, irregular family life, long working hours, and excessive workload can affect the job satisfaction of healthcare employees [11]. There are three areas of harmony. These are the work environment, desiring to be loved/sexuality, and the social environment. There is a relationship between leisure time and life satisfaction, and between job satisfaction and life satisfaction [12]. Sexual life satisfaction impacts couples’ married lives [13]. There is a significant relationship between job satisfaction and work–life balance [14]. Life satisfaction represents a key indicator for psychological health and psychological well-being [15]. The literature shows that naturally occurring increases in sexual satisfaction over time are linked with corresponding increases in life satisfaction [16], and also that incompatibility and dissatisfaction problems in one area can affect other areas. Although there are some studies linking job satisfaction and life satisfaction [11,12], there is no research examining the mediating role of sexual satisfaction and communication skills between job and life satisfaction. The present study contributes to the literature by examining whether satisfaction in terms of sexual and communication skills are associated with changes in life satisfaction and job satisfaction. This shows the originality of the current research. Therefore, the purpose of the present study is to examine the relationship between job and life satisfaction and to test the mediating role of communication skills and sex satisfaction between job and life satisfaction of healthcare employees.

## 2. Literature Review and Theoretical Background

### 2.1. Job Satisfaction

The Hawthorne studies of the late 1920s and early 1930s were the first to introduce the concept of job satisfaction [17]. Job satisfaction refers not just how happy employees are at work, but also their psychological well-being, including their sense of identity, health, and overall happiness. There are two categories of factors that affect job satisfaction. Intrinsic factors include achievement, recognition for achievement, features of the work, responsibility, and individual growth. Policies and administration, working conditions, supervision, status, interpersonal relationships and communication, salary, security, and personal life are some extrinsic motivational factors related to jobs [8]. If motivation is reduced, stress may be increased [18]. The literature also demonstrates that there is a relationship between job satisfaction and stress in workplaces [19,20,21,22].

### 2.2. Life Satisfaction

Life satisfaction refers to one’s happiness with one’s health and meaningful contribution to society, as well as one’s logical evaluation of one’s life and quality of life. It also encompasses psychological and emotional well-being [23]. Life satisfaction is one of the many overlapping facets of subjective well-being. In fact, it is a cognitive evaluation of the overall quality of one’s life. It is used to represent the concepts of “declared happiness” or “subjective well-being” relating to a cognitive evaluation of the overall quality of an individual’s life and their positive evaluation of their whole life [24]. Life satisfaction is defined as an evaluation made regarding the interaction between what a person has or wants to have [25].

### 2.3. Communication Skills

Communication skills are the cognitive and behavioural competencies of individuals to establish positive interpersonal relationships [25]. Sexual satisfaction refers to a positive subjective evaluation of one’s sexual relationship. Sexual satisfaction is important for relationship satisfaction and general well-being. It is thought that marital satisfaction, voluntary communication, love and intimacy, social support, and sexual communication affect interpersonal communication. In the literature, it has been determined that sexual satisfaction is higher in interpersonal relationships in which relationship satisfaction and communication quality are good [26].

### 2.4. Sexual Satisfaction

Sexual satisfaction is an important factor of sexual health and right, and also a result of sexual well-being [27]. Sexual satisfaction is the subjective responses that result from the positive and negative evaluation of the individual’s components of sexual intercourse such as frequency, satisfaction, touching, and avoidance. It is the level of happiness evaluated in terms of biological, social, and psychological aspects [26].

### 2.5. The Link between Job and Life Satisfaction

In the literature, it is mentioned that there are three basic needs: autonomy, competence, and relatedness for people to develop and maintain their lives functionally [28]. These needs, which are expected to be met in personal life and business life, affect individuals’ satisfaction with life. There are many factors that affect life and job satisfaction, such as personal satisfaction with life, feeling that life is worthwhile, reaching goals, positive personality, seeing oneself physically well, economic comfort, and sociability. In particular, when people work in jobs they love and have motivation to work, this increases satisfaction in many areas of life [8]. In the literature, it is stated that having a bachelor’s degree, being married, and having children can increase job satisfaction.

At the same time, it has been determined that working for many years in a profession, or institution, as well as work based on orders, affects job satisfaction. For example, employees who are very happy with their job have been shown to be more productive, creative, and stay in a work organization for longer periods of time [29]. Life satisfaction and job satisfaction are one of the most important reasons for increased productivity and creativity as a result of happiness. In this study, we aimed to evaluate the relationship between the job satisfaction and life satisfaction of health workers. Thus, we tested the link between the job and life satisfaction of healthcare employees (H1).

### 2.6. The Link between Job Satisfaction and Communication Skills

Job satisfaction is one of the most commonly examined topics by researchers attempting to study employee behaviour in the workplace. A high degree of job satisfaction among healthcare employees correlates with a good quality of patient care [30]. Work satisfaction is often measured by an employee’s attitude toward their work environment and perceived level of stress pressure [9]. Communication is an important factor in job satisfaction. Thus, ensuring organizational integrity, probity, and effective communication is necessary for an organization to be more successful. An organization can only achieve its goals with dedicated employees and high job satisfaction. In particular, the satisfaction of health workers with their jobs, ability to communicate clearly with team members and managers, and their dedication to their work can benefit both the institution and the patients [31]. Using communication skills and a high level of job satisfaction are directly linked to a much better quality of services and healthcare [30]. This is closely related with communication of employees in healthcare organizations. Thus, we tested the relationship between the job satisfaction and communication skills of healthcare employers (H2).

### 2.7. The Link between Communication and Life Satisfaction

Life satisfaction is one of the main components of subjective well-being. It is a process that leads to discussion of the commitment, self-devotion, positive effects, satisfaction, and help that make life meaningful [32]. The communication of individuals as social beings, with each other and within societies, is another of the important factors affecting well-being [33]. Therefore, we studied the relationship between the communication skills and life satisfaction of healthcare employers.

One of the main components of subjective well-being is life satisfaction. This process brings in discussion commitment, self-devotion, positive effect, satisfaction, and help that make the life meaningful [32]. The communication of individuals, as social beings, with each other within societies, is one of the important factors affecting well-being as well [33]. Thus, the relationship between communication skills and life satisfaction of healthcare employees was studied. (H3).

### 2.8. The Link between Job, Life, Communication, and Sexual Satisfaction

In general, there are three areas of harmony in life: “work environment”, “love-to be loved-sexual area”, and “social environment”. These areas are related to each other. Incompatibility and dissatisfaction in one area can also affect other areas. The problems that individuals experience in their sexual lives and in the family cause them to be unhappy in their business and social relationships [34]. Therefore, we studied the relationship between sexual satisfaction and life satisfaction of healthcare employees (H4).

Communication skill is the cognitive and behavioural competency of individuals to establish positive interpersonal relationships. It is thought that marital satisfaction, voluntary communication, love and intimacy, social support, and sexual communication affect sexuality in interpersonal communication. One of the most important parts of a fulfilling life is having a happy sex life. The meaning of a happy sexual life varies from person to person; that is, it is subjective, as everyone’s sexual expectations, needs, and sexual desires are different from each other [35]. Research indicates that problems occurring in one of the main factors of society may have effects on a person’s life, such as sexual relationships [36]. Therefore, we studied the relationship between job satisfaction and sexual satisfaction of healthcare employees (H5).

It is well-known that regular working hours and both psychological and physical health can be improved by quality sleep. Sleep and sexual life problems can be caused by the shift work of health workers. Good sex and good sleep are significantly related [37]. Quality sleep is provided by a good sexual life. The list of sleep-inducing manoeuvres seems to include sex. In total, 32% of 866 women who reported masturbating in three months did so to help them sleep [38]. More than 50% of men and women out of 750 showed improved sleep quality after having sex [39]. The lack of regular bedtime for healthcare employees can also affect their sexual health and satisfaction. Thus, we investigated the mediating role of communication skills and sexual satisfaction between job satisfaction and life satisfaction of healthcare employees (H6).

## 3. Materials and Methods

### 3.1. Data Collection and Procedures

The data were collected by using three scales from the healthcare department (ethical permission number: E-20478486-050.04.04-268043). We directly collected data by interviewing participants in the hospital. We first provided information to the participants about the variables to be measured (sex, life, job satisfaction, and communication), and then we made the constant signed by participants who were volunteer to interview and fill the scales.

Hospital workers experience job stress and psychological problems [40]. These problems may affect employee job and life satisfaction. Thus, we used a university hospital as the sample for the current study. A total of 1167 healthcare employees worked in the university hospital studied in Turkey between April and August 2021, of which 394 employees voluntarily participated in the study. The response rate was 33.8%. The sample size was selected based on Comrey and Lee (1992)’s inferential statistics, in which a sample above 300 is considered to be good [41].

### 3.2. Measures

The survey was divided into five sections, the first of which was about the sample characteristics of the participants. The second measure concerned job satisfaction, which was established by Brayfield and Rothe (1951) [42] and reduced by Judge, Locke, Durham, and Kluger (1998) [43]. The validity–reliability issue studied by Başol and Çömlekçi in Turkey in 2020 was also investigated [44]. The job satisfaction scale is made up of five elements and one factor. The next part assessed subjective life satisfaction using five items with a one-dimensional framework created by Diener, Emmons, Larsen, and Griffin (1985) [45]. The Golombok Rust Sexual Satisfaction Scale (GRISS) is another assessment instrument. It is a brief 28-item questionnaire designed to assess the presence and severity of sexual dysfunction [46].

The last section concerned participants’ communication skills, including 7 items developed by Joyce, Steenbergh, and Scher (2010) [47]. A five-point Likert scale was used (1: Strongly Disagree; 5: Strongly Agree).

### 3.3. Analytical Method

**Figure 1:**
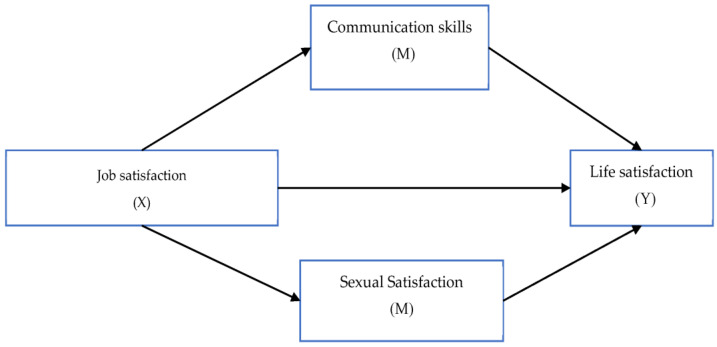
Research model.

Structural Equation Modelling-AMOS was used to examine the relationship between variables. The hypothesis model is presented in Figure 1.

Our hypotheses are as follows:

**H1.**  *Job satisfaction and life satisfaction perceptions of healthcare employees have a positive relationship.*

**H2.**  *Job satisfaction and communication skill perceptions of healthcare employees have a positive relationship.*

**H3.**  *Communication skills and life satisfaction perceptions of healthcare employees have a positive relationship.*

**H4.**  *Sexual satisfaction and life satisfaction perceptions of healthcare employees have a positive relationship.*

**H5.**  *Job satisfaction and sexual satisfaction perceptions of healthcare employees have a positive relationship.*

**H6.**  *Communication skills and sexual satisfaction have a mediating role between job satisfaction and life satisfaction perceptions of healthcare employees.*

## 4. Results

The sociodemographic characteristics of the participants are presented in Table 1.

Many statistical factors must be examined before examining the data to verify that they are distributed normally. To use the Structural Equation Model, the data distribution must be normal. The values of skewness and kurtosis must be between +1 and −1 [48] to show that the data are distributed normally. Skewness and kurtosis values were between +1 and −1 in the current study. To begin, we utilized correlation analysis to identify the relationship between variables in the research model. Table 2 displays the results.

Table 2 demonstrates that there is a strong association between factors. Mediators, according to Baron and Kenny (1986), mediate any associated connection between internal and external variables [49]. Table 3 depicts the modelling route of the direct effect of job satisfaction on life satisfaction, which implies a substantial direct effect in order to examine the mediation effect of communication skills. Table 3 demonstrates that job satisfaction has a significant direct influence on life satisfaction (*p* < 0.05). Therefore, Hypothesis H1 is accepted.

**Figure 2:**
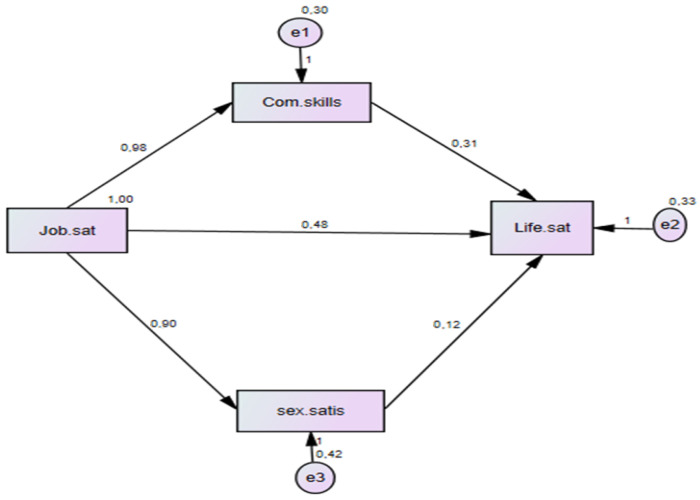
Regression coefficient between all constructs.

The value of the beta coefficient for life satisfaction is expected to decrease when the mediating variable of communication skills is added into the model, meaning that the direct effect of job satisfaction on life satisfaction is lessened. Figure 2 displays the research model, which employs communication skills as a mediator. 

The value for CMIN (χ^2^) is 4.45; *p* = 0.000; RMSEA = 0.064; RMR = 0.072; CFI = 0.984; IFI = 0.984; and GFI is 0.967. From Table 4 below, it can be seen that the value of beta coefficient linking job satisfaction to life satisfaction reduced from 0.90 to 0.48. In this case, job satisfaction was both significant in its direct effect on life satisfaction and indirectly significant in its effect on life satisfaction through the mediator variables; namely communication skills and sexual satisfaction.

As seen on Table 4, job satisfaction has a positive impact on both sexual satisfaction and communication skills (*p* < 0.05). Therefore, H2 and H4 are accepted. Moreover, it can be assumed that communication skills and sexual life can directly influence life satisfaction (*p* < 0.05). Therefore, H3 and H5 are also accepted as the direct effect of job satisfaction on career success is still partially significant even after communication skills and sexual satisfaction enter the model, and though the beta coefficient for job satisfaction is reduced from 0.90 to 0.48. These results show that there is a partial mediation impact of communication skills between job satisfaction and life satisfaction. Therefore, H6 is accepted.

## 5. Discussion

The aim of this study was to determine the relationship between job satisfaction, life satisfaction, communication skills, and sexual satisfaction of healthcare employees. Thus, we tested basically six hypotheses. The present research confirmed that job satisfaction positively relates to life satisfaction (H1) and communication skills (H2), communication skills is positively associated with life satisfaction (H3), sexual satisfaction positively relates with life satisfaction (H4), job satisfaction positively relates with sexual satisfaction (H5), and both communication skills and sexual satisfaction act as mediators between job and life satisfaction (H6).

Firstly, we tested the link between job and life satisfaction (H1) and found that the direct effect of job satisfaction on life satisfaction was significant. Some studies in literature supported this finding [50,51,52,53,54]. Increasing job satisfaction can promote subjective well-being and life satisfaction [51], as well as quality of life and marital satisfaction [32,52]. Job satisfaction is a positive predictor and has an impact on the life satisfaction of healthcare employees [53]. Secondly, we studied the relationship between job satisfaction and communication skills in healthcare employees (H2).

Communication is associated with personal beliefs and values used to share ideas and gain new information. Because humans enjoy getting to know and forming ties with one another, the inability to create personal relationships with one another may result in dissatisfaction, loneliness, and sadness [54]. According to Parson and Stonestreet, communication skills and capacity to listen, communicate expectations effectively, and offer feedback have an important effect on the job satisfaction of healthcare personnel [55].

In Slovakia, the biggest problems in work environments were identified as leadership and direct communication problems with superiors [56]. This causes a high rate of burnout of medical staff and low job satisfaction. In line with previous studies [56,57,58], this study found that healthcare employees were satisfied with communication skills. Similar to the updated research in the literature [59], current research confirms that communication is a very important aspect of job satisfaction in healthcare organizations. Communication skills such as continuous information exchange, effective idea expression, transparency and openness in communication, or the utilization of varied approaches all contribute to increased job satisfaction. The lack of reliable interaction may damage the safety of patients and the quality of care; therefore, it is important to ensure effective and reliable communication and to develop and sustain communication skills in the clinical setting [60]. Thirdly, we evaluated the impact of communication skills on healthcare employees’ life satisfaction (H3). The findings showed that communication skills play a role in healthcare employees’ life satisfaction. This result is in line with many studies documenting links between communication and life satisfaction [61,62,63]. This relationship has been confirmed in the literature. It is, therefore, important for healthcare employees to explore perceptions of their life satisfaction and enhance their communication skills. Fourth, we analysed the relationship between sexual satisfaction and life satisfaction in healthcare employees (H4). With respect to the previous literature in this area, some studies have shown that sexual satisfaction is positively associated with life satisfaction [64,65]. A decline in sexuality was associated with worse life satisfaction and quality [66] and life satisfaction was associated with satisfaction with sex life [67]. Fifth, we examined the relationship between job satisfaction and sexual satisfaction in healthcare employees (H5).

Although work and family life are separate areas, they are intertwined and have a strong relationship and interaction [68]. Therefore, the roles of both fields affect each other. Particularly because of industrialization, working life takes up more time than family life, and the roles of work dominate the roles of family life [69,70]. Because work and family life affect each other, it is accepted that work–family life conflict and balance are a two-way interaction. Work–family conflict causes stress, burnout, low life and marital satisfaction, and various health problems (such as sexual dysfunctions) for the individual. Work–family conflict stems from reasons such as irregular working hours, working conditions, quality time spent with family, spousal support, communication, and sexual life [12,71].

According to previous studies, there is a negative and meaningful link between job satisfaction and conflict between work and family life [72,73]. Previous sexual issues are a major predictor of decreased satisfaction with work and increased workplace stress [74]. This is closely related to sexual satisfaction. If an employee is satisfied with their job, they may experience sexual satisfaction, or vice versa, because job satisfaction is a measure of overall well-being, which is important for sexual satisfaction [75]; it was also reported in the literature that sexual satisfaction leads to higher well-being [76].

Finally, we tested the mediating role of communication skills and sexual satisfaction between job satisfaction and life satisfaction in healthcare employees. The mediating effect of communication skills and sexual satisfaction between job and life satisfaction is novel. This finding suggests that when employees are provided with an environment conducive to well-being, they feel themselves relax both physically and mentally, which are two main factors that affect sex satisfaction, resulting in higher job and life satisfaction [32,51].

## 6. Conclusions

Life and job satisfaction are popular topics in the literature, particularly in healthcare organizations due to the hyper-dynamic environment [77]. Despite the rising amount of literature on job satisfaction, few studies have directly investigated the influence of sexual satisfaction on job satisfaction.

The relationship between life satisfaction, job satisfaction, and life orientation in a group of active nurses and midwives was assessed [62]. Sex satisfaction is one of the most important factors for relationships between couples [78]. It was revealed that life and job satisfaction decreased with the level of occupational burnout of nurses and midwives. Satisfaction with sex life was positively associated with life satisfaction [67] because reduced worker productivity and an increase in the prevalence of physiological and physical health issues reduce job and life satisfaction [79]. Most crucially for our research, sex satisfaction was connected to both job and life satisfaction.

### 6.1. Implications

Hospitals are institutions that provide 24/7 uninterrupted service, include different working systems, and have an excessive workload. Due to these features, hospitals can be considered to be places where employees’ work stress and work–family conflict are seen more often. This research can provide contributions to healthcare organizations. We found that there is a strong relationship between job, life, and sexual satisfaction and communication skills. These are very important findings for healthcare organizations’ managers. They should pay attention to these factors to maximize their success and increase employee motivation to create a positive organization climate which is critically important in this hyper-dynamic environment.

In terms of the effects of health employees’ satisfaction with work, this study verifies earlier results that when they are pleased with their jobs, their performance and life satisfaction improve. Additionally, when health staff are pleased with their working environment and have good communication skills, life satisfaction and sexual satisfaction are often increased. Thus, a serious source of stress for employees in the health sector is also related to the management of patient data and how these data are transmitted and managed within hospitals by hospital staff [80].

Healthcare organizations, irrespective of size, should endeavour to consider factors such as life satisfaction, sexual satisfaction, and relationships which are relevant to job satisfaction and employee performance. Organizations should strive to satisfy employee needs to enable them to perform effectively. Satisfying these needs will not only lead to job satisfaction but also to life satisfaction, bearing in mind that to some extent, a satisfied employee is a happy citizen. As a result, if policymakers in the health sector establish programs that enhance health practitioners’ satisfaction with their jobs, it will benefit not only employees’ health, but additionally the health of the public and patients.

### 6.2. Limitations

Various limitations to this work are outlined here. The first is that the study is geographically limited to Turkey, although some other studies have been carried out on adult populations [39], among marital couples [54], and in the public sector [72]. Future research may focus on these variables in different sectors and regions of Turkey.

The second limitation is gender. We did not test the relationship between gender and the variables of the current study. There are some studies on gender and experiences, satisfactions in the local literature [81], and between job satisfaction and gender in the overall literature [82,83]. Future research may test the relationship between gender and some variables of current research. Moreover, Turkey is one of the most conservative countries with respect to sexual and gender minorities. Sexuality is still a stigmatized topic in Turkey. Therefore, it is hard to obtain responses from participants about their sexual life.

The third limitation is the relatively small sample of respondents (*n* = 394) given the total number of health employees in Turkey. Future researchers may apply the current model to larger samples in other countries. The fourth limitation is the relationship between the sociodemographic characteristics of the participants and job, sex, and life satisfaction, particularly the link between the role of participants (employee and managers) and job, sex, and life satisfaction. Future researchers may test this link.

## Figures and Tables

**Table 1:** Demographics of the samples.

Demographics	*n* (%)
Gender	
Female	266 (67.4)
Male	128 (32.6)
Age	
22–30	111 (28.2)
31–40	192 (48.7)
41–54	91 (23.1)
Education	
High school	58 (14.6)
Bachelor’s degree	263 (66.7)
Master’s or PhD degree	73 (18.7)
Employment status	
Employee	304 (77.2)
Manager	90 (22.8)
Marriage status	
Single	121 (30.8)
Married	273 (69.2)
Total	394 (100.0)

**Table 2:** Correlation between variables.

Correlations					
		Job Satisfaction	Life Satisfaction	Communication Skills	Sexual Satisfaction
Job Satisfaction	Pearson Correlation	1			
	Sig. (2-tailed)				
	*N*	394			
Life Satisfaction	Pearson Correlation	0.754 **	1		
	Sig. (2-tailed)	0			
	*N*	394	394		
Communication Skills	Pearson Correlation	0.797 **	0.736 **	1	
	Sig. (2-tailed)	0	0		
	*N*	394	394	394	
Sexual Satisfaction	Pearson Correlation	0.742 **	0.771 **	0.705 **	1
	Sig. (2-tailed)	0	0	0	
	*N*	394	394	394	394

** Correlation is significant at the 0.01 level (2-tailed).

**Table 3:** Direct effect of job satisfaction on life satisfaction.

			Estimate	S.E.	C.R.	*p*	CMIN/DF	CFI	GFI	IFI	RMSEA	RMR
H_1_: Life satisfaction	←	Job satisfaction	0.796	0.031	29.6024	***	2.217	980	974	980	0.043	0.047

*** is significantly different from zero at the 0.001 level (two-tailed). Note: S.E. = standard error; C.R. = critical ratio; *p* = probability value; CMIN/DF = X2 value/degree of freedom; CFI = compare goodness-of-fit index; GFI = goodness-of-fit index; IFI = incremental goodness-of-fit index; RMSEA =root mean square error of approximation; RMR = root-mean-squared residual.

**Table 4:** Hypotheses testing.

Direct Paths			Estimate	S.E.	C.R.	*p*
H2: communication skills	←	job satisfaction	0.984	0.027	35,785	***
H3: life satisfaction	←	communication skills	0.309	0.054	5771	***
H4: sexual satisfaction	←	job satisfaction	0.899	0.033	27,518	***
H5: life satisfaction	←	sexual satisfaction	0.121	0.045	2680	0.007
H6: life satisfaction	←	job satisfaction*sex satisfaction	0.483	0.073	6663	***

*** is significantly different from zero at the 0.001 level (two-tailed).

## Data Availability

The data are not publicly available due to privacy and ethical restrictions.
